# Editorial: Insights in genome editing in human health and disease 2023/2024

**DOI:** 10.3389/fgeed.2025.1697828

**Published:** 2025-09-22

**Authors:** Baisong Lu, Haiwei Mou, Chen Liang

**Affiliations:** ^1^ Wake Forest Institute for Regenerative Medicine, Wake Forest University School of Medicine, Winston-Salem, NC, United States; ^2^ The Wistar Institute Melanoma Research Center, The Wistar Institute, Philadelphia, PA, United States; ^3^ Lady Davis Institute, Department of Medicine, McGill University, Montreal, QC, Canada

**Keywords:** CRISPR/Cas9, genome editing, gene conversion, homologous recombination, public perception, immune-evasive hESCs

In 2023, FDA approved one CRISPR/Cas9‐based gene therapy to treat sickle cell disease, which marks a significant milestone in the translation of genome editing technologies into clinical therapeutics. In light of this exciting advancement, we launched a Research Topic aimed at gaining new insights, reporting novel developments and recent discoveries, discussing current challenges, and exploring future perspectives in the field of Genome Editing in Human Health and Disease. This topic collected four publications: two original research articles, one review article, and one systematic review. These papers address a range of important issues, including how to cope with the underappreciated impact of genomic homologous sequences on editing outcomes, the challenges posed by immune rejection, a comprehensive overview of genome editing technologies, and public perceptions surrounding these innovations.

In the study by Lagas et al., when the authors attempted to create *GBA1* knockout iPSC lines, they found that the Insertion and Deletion (INDEL) rate was low, and majority of the edited alleles were the results of gene conversion (Chen et al., 2007) of a pseudogene *GBAP1*, which is 96% identical to and 16 kb downstream of *GBA1*. CRISPR/Cas9-mediated genome editing was previously found to increase gene conversion using genomic homologous sequences as the template to repair DNA damages via homologous recombination without crossover (Javidi-Parsijani et al., 2020). Thus, this study reports another example of CRISPR/Cas9-mediated gene conversion. The authors then used single-stranded oligodeoxynucleotide (ssODN) donors to compete with the endogenous pseudogene *GBAP1* and successfully obtained iPSC line with *GBA1* knockout. This study provides a method to improve the efficiency of CRISPR/Cas9-mediated gene knockout when highly homologous sequences are present in the genome.

Frederiksen et al. attempted to create immune-evasive hESCs using CRISPR/Cas9 to knock out *B2M* and *CIITA* genes, encoding the major histocompatibility complexes I- and II respectively, in human embryonic stem cell lines (hESCs). In addition, they also overexpressed the mouse CD47, a “do not eat me” signal (Tsai and Discher, 2008), in hESCs. They found that the genetically modified hESCs were still rejected after being transplanted into immune competent mice. Their results showed that these modifications are insufficient to prevent rejection in an immune-competent and xenogeneic context.

Azeez et al. provided a comprehensive review on the development of CRISPR/Cas technology since the publication of Doudna and Charpentier’s seminal work on CRISPR/Cas9 in 2012 (Jinek et al., 2012). They discussed the diverse types of Cas endonucleases, the various genome editing technologies derived from these CRISPR/Cas systems, the physical, chemical and biological strategies of CRISPR/Cas delivery, and the applications, especially the clinical application of these technologies. Over 100 CRISPR/Cas-related clinical trials were recorded. This comprehensive review is a great resource for researchers new to the field as well as experts already in the genome editing field.

Ramos et al. systematically reviewed public perceptions on genome modification before (pre-CRISPR) and after 2013 (CRISPR). The authors discussed 53 primary publications (1987–2020) of surveys addressing public attitudes toward applications of genetic modifications in humans and animals from different countries in four continents. An interesting finding is that whether before or after the discovery of the CRISPR technology, it is highly acceptable to the public using gene modifications for disease treatment and prevention in humans, whereas the public are opposed to using them for enhancement. The public accept somatic gene editing more than gene editing in germlines.

In summary, these four papers have covered very important aspects of the CRISPR technology, from methodology of improving genome editing efficiency in special situations, to possible applications in preventing immune rejections, to public perspective on the application of these technologies. We hope that these papers will promote the further development and application of the CRISPR technology.

## References

[B1] Chen J. M. Cooper D. N. Chuzhanova N. Ferec C. Patrinos G. P. (2007). Gene conversion: mechanisms, evolution and human disease. Nat. Rev. Genet. 8, 762–775. 10.1038/nrg2193 17846636

[B2] Javidi-Parsijani P. Lyu P. Makani V. Sarhan W. M. Yoo K. W. El-Korashi L. (2020). CRISPR/Cas9 increases mitotic gene conversion in human cells. Gene Ther. 27, 281–296. 10.1038/s41434-020-0126-z 32020049

[B3] Jinek M. Chylinski K. Fonfara I. Hauer M. Doudna J. A. Charpentier E. (2012). A programmable dual-RNA-guided DNA endonuclease in adaptive bacterial immunity. Science 337, 816–821. 10.1126/science.1225829 22745249 PMC6286148

[B4] Tsai R. K. Discher D. E. (2008). Inhibition of “self” engulfment through deactivation of myosin-II at the phagocytic synapse between human cells. J. Cell Biol. 180, 989–1003. 10.1083/jcb.200708043 18332220 PMC2265407

